# Filling the glass: Effects of a positive psychology intervention on executive task performance in chronic pain patients

**DOI:** 10.1002/ejp.1214

**Published:** 2018-04-14

**Authors:** J.J.L.M. Boselie, L.M.G. Vancleef, M.L. Peters

**Affiliations:** ^1^ Clinical Psychological Science Maastricht University Maastricht The Netherlands

## Abstract

**Background:**

Chronic pain is associated with emotional problems as well as difficulties in cognitive functioning. Prior experimental studies have shown that optimism, the tendency to expect that good things happen in the future, and positive emotions can counteract pain‐induced task performance deficits in healthy participants. More specifically, induced optimism was found to buffer against the negative effects of experimental pain on executive functioning. This clinical experiment examined whether this beneficial effect can be extended to a chronic pain population.

**Methods:**

Patients (*N* = 122) were randomized to a positive psychology Internet‐based intervention (PPI;* n *=* *74) or a waiting list control condition (WLC;* n *=* *48). The PPI consisted of positive psychology exercises that particularly target optimism, positive emotions and self‐compassion.

**Results:**

Results demonstrated that patients in the PPI condition scored higher on happiness, optimism, positive future expectancies, positive affect, self‐compassion and ability to live a desired life despite pain, and scored lower on pain catastrophizing, depression and anxiety compared to patients in the WLC condition. However, executive task performance did not improve following completion of the PPI, compared to the WLC condition.

**Conclusions:**

Despite the lack of evidence that positive emotions and optimism can improve executive task performance in chronic pain patients, this study did convincingly demonstrate that it is possible to increase positive emotions and optimism in chronic pain patients with an online positive psychology intervention. It is imperative to further explore amendable psychological factors that may reduce the negative impact of pain on executive functioning.

**Significance:**

We demonstrated that an Internet‐based positive psychology intervention strengthens optimism and positive emotions in chronic pain patients. These emotional improvements are not associated with improved executive task performance. As pain itself often cannot be relieved, it is imperative to have techniques to reduce the burden of living with chronic pain.

## 1 Introduction

Chronic pain causes marked behavioural and emotional changes, impacting quality of life (Breivik et al., 2006; Hart et al., 2000). Persistent pain can also lead to difficulties in executive functioning (Moriarty et al., 2011; Berryman et al., 2013, 2014), with some patients even stating that these deficits are more disturbing and disabling than the pain experience itself, especially when it compromises a patient's family live, their professional work productivity and employment status (Stewart et al., 2003; Breivik et al., 2006; Kowal et al., 2012). These pain‐related deteriorations in executive functioning are thought to reflect the capacity of pain to consume attentional resources needed for accurate task performance (Legrain et al., 2009). Attention is a cognitive process that facilitates the processing of information that enables executive functioning. Executive functioning comprises of multiple cognitive processes (Banich, 2009) and is typically decomposed in three core domains that represent specific functions, that is inhibition, updating and shifting (Miyake et al., 2000). Inhibition refers to the ability to purposely inhibit automatic prepotent responses. An example is refraining from using the clutch when driving an automatic car (Miyake et al., 2000). Updating is being able to actively update, manipulate and monitor relevant information held in working memory. For instance, performing mental math requires keeping partial results in mind while solving the problem (Morris and Jones, 1990). Shifting is the ability to switch flexibly and fluently between mental sets and tasks (Gilbert and Burgess 2008), enabling a person for example to switch between different languages in a conversation.

Research has shown that experimental (Buhle and Wager, 2010; Moore et al., 2012; Van Ryckeghem et al., 2012) and persistent pain (Moriarty et al., 2011; Berryman et al., 2013, 2014; Moore et al., 2013; Keogh et al., 2014) impair the performance on tasks that tap into these executive functions. Optimism (i.e. the tendency to expect that good things will happen in the future Carver et al., 2010) and positive affect may improve executive functioning. Drawn from the broaden‐and‐build theory (Fredrickson, 2001), positive affect may (re) direct an individual's attention (i.e. the broaden hypothesis) towards accurate task performance and/or increase cognitive resources (i.e. the build hypothesis). Moreover, increasing optimism and positive affect, by means of writing and visualizing about a positive future, was previously shown to reduce pain‐induced deteriorations in updating (Boselie et al., 2014) and shifting (Boselie et al., 2017) task performance. However, these studies used experimental pain and were conducted in healthy patients. We therefore set up a clinical experiment to examine whether the protective effects of optimism and positive affect on executive functioning impairments extend to a chronic pain population. Pain patients completed an 8‐week Internet‐based positive psychology intervention (PPI) that was previously found to significantly increase optimism and positive emotions in chronic pain patients (Peters et al., 2017). We hypothesized that the positive psychology intervention, compared to a waiting list condition, would lead to (1) an increase in positive and decrease in negative states and traits and (2) improved executive (i.e. updating, shifting and inhibition) task performance.

## 2 Methods

### 2.1 Patients

Recruitment took place from January to August 2014 through an advertisement in a magazine and on the website of the Dutch Society for Fibromyalgia patients. Patients could sign up for the study by contacting the researcher (email) or leave their contact information on a response form that was embedded on the website that was developed for this study. In total, 180 patients signed up for the study and were contacted by phone for a screening interview to check their compliance with the inclusion criteria (see Supporting Information Table S1 for these criteria). Following screening, 53 patients were excluded: 16 because they could not be reached and 37 because they did not fulfil the inclusion criteria. Patients were informed via email whether they were included in the study and were subsequently randomized into the positive psychology intervention (PPI) or waiting list condition (WLC). Randomization occurred by a 2:1 ratio, with more patients being allocated to the PPI condition so that more patients would benefit from the intervention. A minimal sample size of 68 patients was determined with G*Power (Faul et al., 2007) with the following parameters: α = 0.05, power = 0.90 and an effect size of 0.40 (as based on prior studies (Peters et al., 2017; Verdejo‐García et al., 2009). The unequal allocation scheme requires that 12.5% more patients (i.e. 77; PPI > 52 and WLC > 26) should be tested to obtain the same statistical precision as with an equal allocation scheme (Korn and Freidlin, 2011). Six (PPI *n* = 3; WLC *n* = 3) patients did not fill out all the pretreatment measures leaving a total of 121 (PPI *n* = 73; WLC *n* = 48) patients in the study.

### 2.2 Measures

#### 2.2.1 Intervention

The positive psychology intervention (PPI) ‘happy despite pain*’* was offered as an 8‐week online intervention, whereby patients performed positive psychology exercises (Peters et al., 2017). Patients received instructions about the exercises via the online platform and conducted each exercise individually at home. The PPI consists of four modules (i.e. (1), self‐compassion, (2) positive focus, (4) savouring and (5) optimism) that were consecutively targeted in weeks 1–7 of the intervention. The PPI intervention does not focus the pain itself, but rather on enhancing resilience factors and trying to let the patients focus on what they value in life, *despite* their pain (Goubert and Trompetter, 2017).

Module 1 targets self‐compassion, which refers to fully accepting oneself, the ability to treat oneself with kindness rather than self‐criticism (Neff and Germer, 2013). For example, patients write a self‐compassion letter to themselves about their perceived shortcomings and failures, but from a friend's perspective. Module 2 requires patients to practice the ‘three good things’ exercise on a daily basis. Patients write down three good things that happened that day and why they happened. This exercise aims to shift the focus from a negative orientation towards a more positive one, by raising awareness for the good things in life (Seligman et al., 2006). Module 3 contains savouring techniques that promote the frequency and intensity of positive experiences in daily life. Patients actively plan pleasant activities, so‐called ‘mini vacations’ of 20 min. In module 4, patients practice the best possible self (BPS) exercise (Peters et al., 2010), which is used to increase optimism. In the BPS exercise, patients write about and imagine an ideal future life despite the pain. Patients select three unique domains from a list of seven life domains (i.e. family, romantic, social, professional, educational life, leisure and hobby, societal commitment and personal growth). Within these three domains, patients formulate concrete and obtainable future (i.e. over 5 years) goals, wishes and expectations (see Supporting Information Fig. S1 for an overview of all the exercises per module). The last week (i.e. week 8) of the PPI aims to prevent relapse, by creating a personalized plan concerning future use of the learned exercises.

Regular contact moments were organized within the 8‐week PPI, to give support, enhance adherence to the programme and to prevent dropout. Patients were contacted by telephone (weeks 1, 3, 5 and 7) and email (weeks 2, 4, 6 and 8). In the semi‐structured telephone calls, patients were asked whether they had questions or had encountered problems regarding the assignments. The mean duration of the telephone support was 10.56 (SD* *= 7.74) min. The semi‐standardized emails encouraged patients to continue with the exercises and to send an email if they encountered problems.

### 2.3 Questionnaires

#### 2.3.1 Optimism

The Life Orientation Test‐Revised (LOT‐R) (Scheier et al., 1994) measured dispositional optimism and consisted of 10 items: three positively phrased items, three negatively phrased items and four filler items. The items were rated on a 5‐point Likert scale, ranging from 1 (strongly disagree) to 5 (strongly agree). The total LOT‐R score was obtained by summation of the scores on the positively phrased items and the reversed scores on the negatively phrased items and ranged from 10 to 30. Higher scores reflect higher levels of dispositional optimism. The LOT‐R has been demonstrated to be a reliable and valid measurement instrument (Scheier et al., 1994) and internal consistency was satisfactory in this study with a Cronbach's alpha of 0.77.

#### 2.3.2 Future expectancies

The Future Expectancies Scale FEX (Hanssen et al., 2013) measured positive and negative future expectancies. It consisted of 20 items that make statements about positive (*n *=* *10) and negative (*n *=* *10) future events. The 20 statements covered five different domains (work, health, personal, social and general). Patients rated the likelihood that they will experience the future events on a 7‐point Likert scale, ranging from 1 (not at all likely to occur) to 7 (extremely likely to occur). Higher scores reflect a higher estimated likelihood of positive (FEX‐Pos) or negative (FEX‐Neg) future events, with scores ranging from 10 to 70. The internal consistency of the FEX subscales has been demonstrated to be satisfactory (Hanssen et al., 2013). Cronbach's alphas in this study for the positive and negative subscale were 0.87 and 0.83, respectively.

#### 2.3.3 Self‐compassion

The Self‐Compassion Scale‐Short Form (SCS‐SF) (Neff, 2003) consisted of 12 items divided over six subscales. These subscales corresponded to the six components of self‐compassion: Self‐Kindness, Self‐Judgment, Common Humanity, Isolation, Mindfulness and Over‐Identification (Neff, 2003). In this study, the items were rated on a 7‐point Likert scale (Wechsler, 1997), ranging from 1 (almost never) to 7 (almost always) and scores ranged from 12 to 84. The total self‐compassion score was computed by adding all subscale scores, after reversing the negative subscale items (i.e. items of the subscales Self‐Judgment, Isolation and Over‐Identification). The SCS–SF demonstrated adequate internal consistency and a near‐perfect correlation with the long‐form SCS (Raes et al., 2011). In this study, internal consistency was good with a Cronbach's alpha of 0.89.

#### 2.3.4 Depression and anxiety

The Hospital Anxiety And Depression Scale (HADS) (Zigmond and Snaith, 1983) measured depressive and anxiety symptoms in medical outpatient clinics. The HADS consisted of 14 items (including six reversed items): seven measured depressive symptoms and seven measured anxiety symptoms. Items were rated on a 4‐point Likert scale ranging from 0 (indicating no depressive or anxiety symptoms) to 3 (indicating a high level of depressive or anxiety symptoms). The total depression or anxiety score was obtained by summation of the scores on the negatively phrased items and the reversed scores on the positively phrased items. Scores for the subscales range from 0 to 21, with scores 0–7 indicating ‘normal’, scores 8–10 indicating ‘mild’, scores 11–14 indicating ‘moderate’ and scores 15–21 indicating ‘severe’ presence of symptoms (Zigmond and Snaith, 1983). The HADS has been demonstrated to be a reliable measurement instrument (Spinhoven et al., 1997). Cronbach's alphas for the anxiety and depression subscale were both 0.82.

#### 2.3.5 Happiness

Happiness was assessed via a single question that inquired about the degree of happiness one feels at this moment. Answers were scored on a 6‐point Likert scale with scores 1 (very unhappy), 2 (unhappy), 3 (more unhappy then happy), 4 (more happy then unhappy), 5 (happy) to 6 (very happy). A higher score reflects greater happiness. Measuring happiness by single‐item questions has been proven to be reliable and valid (Abdel‐Khalek, 2006).

#### 2.3.6 Pain Catastrophizing

The Pain Catastrophizing Scale (PCS) (Sullivan et al., 1995) consisted of 13 items reflecting catastrophic thoughts and emotions regarding pain. Patients indicated to what degree they experienced each of 13 stated thoughts and feelings while experiencing pain on a 5‐point Likert scale, ranging from 0 (not at all) to 4 (all the time). The total PCS score was obtained by summing the responses of all the 13 items (scores range from 0 to 52). Higher scores on the PCS indicate greater pain catastrophizing. Reliability and validity of the PCS total and subscales were adequate (Severeijns et al., 2002). In this study, internal consistency was good with a Cronbach's alpha of 0.93.

#### 2.3.7 Affect

The Positive and Negative Affect Schedule (PANAS) (Watson et al., 1988) consisted of 20 items that measure positive (PA, 10 items) and negative (NA, 10 items) affect. Patients indicated the degree to which a certain feeling was present at that moment on a 5‐point Likert scale ranging from 1 (not at all) to 5 (extremely). Subscale scores ranged from 10 to 50, with higher scores on NA items reflecting higher levels of emotional distress. In contrast, high PA scores correspond to experiencing more pleasurable feelings. The PANAS subscales have been demonstrated to be valid and reliable (Crawford and Henry, 2004). Cronbach's alphas in this study for the positive and negative subscale were 0.87 and 0.88, respectively.

#### 2.3.8 Pain

Three Visual Analogue Scales (VASs) were administered to measure the patient's experience of pain at that moment, namely experienced pain intensity, burden of pain and ability to live a desired life despite the pain. Each VAS was anchored 0 (no pain at all/no burden at all/not at all able) to 100 (extreme pain/extreme burden/completely able).

#### 2.3.9 Pain disability

The Pain Disability Index (PDI) (Pollard, 1984) measured the impact that pain has on the ability of a person to participate in seven categories of life activities: family and home responsibilities, recreation, social activity, occupation, sexual behaviour, self‐care and life‐support activity. Patients indicated the level of disability that they typically experience on an 11‐point Likert scale with 0 (no disability) to 10 (total disability). The total PDI score was obtained by the summation of the seven items and ranged from 0 to 70, with greater scores indicating greater disability due to pain. The PDI has shown to be a valid and reliable measure of pain‐related disability (Tait et al., 1990). In this study, internal consistency was good with a Cronbach's alpha of 0.84.

#### 2.3.10 Task experience

Four Visual Analogue Scales (VASs) measured the patients experience concerning the execution of the executive functioning tasks, namely experienced fatigue, difficulty, level of pain during task execution and ability to concentrate on task execution. Each VAS was anchored 0 (not fatiguing at all/not at all difficult/no pain at all/not at all difficult to concentrate) to 100 (extremely fatiguing/extremely difficult/extremely painful/extremely difficult to concentrate).

### 2.4 Executive functioning

In selecting the executive functioning tests, we aimed to include paradigms that are regarded as standard tests for the three core executive functions that are often proposed, namely inhibition, updating and shifting (Miyake et al., 2000). We specifically included tasks that had been used in prior studies examining executive functioning in relation to pain (Buhle and Wager, 2010; Moriarty et al., 2011; Moore et al., 2012, 2013; Van Ryckeghem et al., 2012; Berryman et al., 2013, 2014; Keogh et al., 2014). Moreover, given the online nature of our study, we selected tasks that could be assessed in an online procedure.

### 2.5 Inhibition

#### 2.5.1 Stop‐signal task

In this reaction time paradigm (Benedict and Zgaljardic, 1998), patients were instructed to respond to a stimulus as quickly as possible (by pressing a button), unless a specific cue (i.e. stop‐signal) was presented in which case the response needs to be inhibited. Hence, patients need to inhibit an initiated motor action when this is suddenly no longer appropriate. Reaction times on so‐called stop‐trials versus go‐trails are used to infer difficulties in inhibition (see below).

The stop‐signal task consisted of 160 trials. Each trial started with a fixation cross that was presented for 500 ms in the middle of the computer screen, followed by either a black arrow (i.e. the go‐stimulus) pointing to the left or right. Patients were required to press the ‘E’ key on the keyboard if the arrow pointed to the left or the ‘U’ key when the arrow pointed towards the right. When the arrow turned red (i.e. stop‐signal), which was the case on 25% of the trials (i.e. 40 trials), patients were expected to inhibit their response and refrain from pressing a button. The time interval between the go‐stimulus and the stop‐signal (i.e. stop‐signal delay) was initially set to 250 ms and then adjusted dynamically depending on the patient's responses during task performance. The stop‐signal delay was increased or decreased by 50 ms on the next stop‐signal trial when subjects succeeded or failed to stop, respectively. Increasing the stop‐signal delay makes it more difficult to inhibit the next stop‐signal trial, while decreasing makes it easier to inhibit the next stop‐signal trial. Adjusting the stop‐signal delay dynamically during task performance ensures that patients have an approximately equal number of failed as successful stop‐trials (Benedict and Zgaljardic, 1998).

The task started with a practice phase (30 go‐trials). Response feedback was given on each trial (‘correct’ or ‘incorrect’). Moreover, patients were urged to respond faster on every trial with a message that appeared below the arrow stimulus, irrespective of their reaction time. The test phase of the task consisted of four blocks containing 40 trials each (i.e. 30 go‐trials and 10 stop‐trials). Patients were allowed to take short self‐paced breaks in between blocks. Task instructions were displayed in the left and right upper corners of the computer screen, ‘press E for left’ and ‘press U for right’, respectively. In the middle bottom part of the computer screen, patients could follow their progress, via an indicator that displayed the number of completed and uncompleted trials for that block (e.g. 1/30). In the test phase, a feedback message was only given when responses on the go‐trials became too slow (i.e. >2 SD above individual cut‐off score, which was calculated on the basis of the mean RT and SD as obtained in the practice phase). This message urged patients to respond faster, preventing patients to use this response slowing in order to improve accuracy on stop‐signal trials (Bialocerkowski, 2008). The total duration of the stop‐signal task was approximately 10 min. The main outcome variable of the task was the stop‐signal RT (SSRT) that is calculated by subtracting the mean stop‐signal delay (i.e. time between the go‐stimulus and the stop‐signal) from the mean RT on correct go‐trials. Higher SSRTs indicates that the patient needed more time to inhibit a response, reflecting less inhibitory control (Benedict and Zgaljardic, 1998).

### 2.6 Updating

#### 2.6.1 Reverse digit span

The reverse digit span originates from the WAIS–III (Wechsler, 1997). A series of digits (1, 2, 3, 4, 6, 7, 8 and 9) of increasing length were presented one‐by‐one on a computer screen, starting with a series of three digits to a maximum of eight digits. After the last digit was presented, patients had to repeat the numbers in the reverse order, by clicking with the left mouse button on the corresponding numbers in a numeric pad that was presented on the bottom part of the computer screen. Following each successful trial, a digit was added to the next trial. The score (i.e. correctly recalled digits) was shown in the upper part of the screen. The task ended when a patient failed to accurately reproduce a series of digits of the same length on two successive trials. The total score was calculated by summing the total amount of digits correctly recalled. The total duration of the reverse digit span was <6 min.

### 2.7 Shifting

#### 2.7.1 Task shifting

The task‐shifting paradigm was used to measure shifting ability. In the shifting task, eight single‐digit numbers (1, 2, 3, 4, 6, 7, 8 and 9) were presented one‐by‐one on a computer screen. Patients needed to follow a discrete task rule on each trial, which can either change (i.e. switch trial) or remain the same (i.e. repetition trials) from one trial to the next. Responses on a switch trial are typically slower and less accurate compared to responses on a repetition trial (i.e. switch costs). See Supporting Information Fig. S2 for an example of two trials. Patients had to follow two task rules, namely indicating whether the target number was higher or lower than 5 and indicating whether the target number was even or odd. One of both task rules (i.e. higher/lower or odd/even) was briefly primed (500 ms) on screen before the presentation of the next target number (Kiesel et al., 2010). The target number was then shown until the patient gave a response. The mapping of response keys to task rules was counterbalanced across patients. Which key corresponded to which answer type (e.g. E = lower/even and U = higher/odd) was simultaneously presented with the target number in order to ensure that patients were aware of the required sorting options.

The shifting task consisted of a practice phase and testing phase. The practice phase included 12 trials, with six higher/lower trials and six odd/even trials. In the testing phase, 192 trials were presented, with 96 switch trials of which 48 were congruent (i.e. trials for which the correct response is mapped to the same key under both task rules Altmann and Gray, 2008). The presentation of target numbers was fixed semi‐randomized to ensure the previously mentioned distribution of switch and congruent trials. Additionally, the same priming cue was not presented for more than four consecutive trials, to prevent an automatic response flow.

The total duration of the shifting task was <15 min. Reaction times (RT) and accuracy were registered. The main dependent variables used in this study were RT switch costs and accuracy switch costs. The RT switch cost score was computed by subtracting RT on repeat trials from switch trials, such that higher positive values indicate that patients were slower to respond to switch trials than to repetition trials. The accuracy switch cost score was computed by subtracting the proportion of correct responses on switch trials from the proportion of correct responses on the repeat trials, such that higher positive values indicate that patients were less accurate on switch trials compared to repetition trials.

#### 2.7.2 Wisconsin card sorting task

We used a modified, computerized Wisconsin card sorting task (WCST) version (Tien et al., 1996). In its conventional form (Heaton et al., 1993), the task can be discontinued when six categories (i.e. a sequence of ten correct responses to one specific sorting rule) are completed. We required that all 128 response cards had to be sorted (Greve et al., 2005).

In the WCST, four stimulus cards were presented on the bottom half of the computer screen and one response card on the upper half of the computer screen. The response card changed every trial; the four stimulus cards remained unchanged (i.e. card 1 with a red triangle, card 2 with two green stars, card 3 with three yellow crosses and card 4 with four blue circles on them). Every response card had three possible attributes: form, number and colour. Every attribute had four possible features: form (triangle, star, cross and circles), number (1, 2, 3 and 4) and colour (red, green, yellow and blue). Patients had to match the response card (128 in total) to one of the four stimulus cards by clicking with the left mouse button on the selected stimulus card. Patients were not informed about the sorting rule (i.e. sorting by form, number or colour). Patients had to find the correct sorting rule by trial and error. Computer feedback (i.e. ‘right’ or ‘wrong’) was displayed on the computer screen after matching the response card to a stimulus card. After ten consecutive correct matches, the sorting rule changed without warning, requiring the patient to shift their cognitive strategy. See Supporting Information Fig. S3 for an example.

The sequence of sorting rules was counterbalanced, but with the restriction that within a counterbalance condition, the sequence of the rules remained the same (e.g. colour, form, number, colour, form, number and so on). Patients received an identical stimulus list in the pre‐ and postmeasurement. The number of categories completed was the main outcome variable. Any time a patient interrupted a sequence of correct responses by making an error, the category of that sorting rule was not obtained. The same sorting rule applied until 10 correct matches with that specific rule was achieved. Task duration was <15 min.

### 2.8 Procedure

The study took place between February 2014 and January 2015. After randomization, patients received an email invitation to complete the online premeasurement, consisting of two sessions. Session 1 contained questionnaires on demographic information, medication use and happiness, and VASs on pain and task perception. Moreover, two executive functioning tasks were administered (i.e. stop‐signal task and WCST). We also administered the 2‐back task (Boselie et al., 2016), but we do not report this data as too many patients (*n *=* *84, 68.9%) scored an accuracy score below chance level (<54% correct, as determined by a binomial calculator). Patients were invited via email to complete session 2 the next day. This session contained the PANAS, PCS, FEX, LOT‐R, HADS, PDI, SCS‐SF, the VASs on task perception and two executive functioning tasks (i.e. shifting task and reverse digit span). We decided to divide the pre‐and postmeasurement in two sessions to avoid fatiguing patients. Patients were instructed to complete the sessions in a quiet environment and to take sufficient self‐paced breaks between the questionnaires or tasks. However, they were requested to always complete a questionnaire or task once they had started.

Following completion of the premeasurement, patients in the PPI condition received their username and password via email to the site where the intervention was hosted. Patients could access the site through the username and password and a six‐digit security code that was provided to their mobile phone or email at every login. The intervention consisted of eight weeks. In the first week, only the exercises of that week could be accessed. The following exercises became consecutively available exactly one week after the previous one. Mean duration of the intervention for intervention completers was 9.9 (SD = 2.1) weeks (range 8–17 weeks). Each online module consisted of information about the topic of that week and practical assignments. Patients were also sent a printed workbook at the start of the intervention that contained a summary of the online information and the corresponding assignments. Patients could also complete the assignments in this workbook and use it as a future reference book. However, it was emphasized that patients should first and foremost complete the assignments online.

After completion of the intervention, patients were invited via email to complete the postmeasurement via Internet. The postmeasurement again consisted of two sessions using the same partitioning of tasks and questionnaires (except demographic information) as during the premeasurement. In the WLC condition, after completion of the premeasurement, patients were informed of the 8‐week waiting period and when they would receive the invitation for their postmeasurement. Patients could start the intervention after completion of the postmeasurement. Patients in WLC condition did not receive the weekly support of an assistant by telephone or email. After completion of the postmeasurement, all patients received a gift (lantern bag) to thank them for participation in the study.

### 2.9 Data analyses

Data were checked for a normal distribution, and reliability analyses were performed on the scores of the premeasurement of the LOT‐R, FEX, PANAS, SCS‐SF, PCS, HADS and the PDI questionnaires. Independent‐samples *t*‐tests were used to check for baseline differences between the conditions (PPI vs. WLC) on questionnaire scores, executive functioning and pain duration and chi‐square tests for categorical variables (demographic characteristics). To examine possible predictors of dropout, a logistic regression analyses were performed with condition (PPI vs. WLC) and all the baseline variables as predictors.

Executive functioning data were screened for outliers. On the reaction time (RT) tasks, RT < 250 ms or 3 × SD above individual mean RT was replaced with the cut‐off value (i.e. 250 or 3 × SD above individual mean RT). Furthermore, incorrect trials were omitted (shifting task 12.0% (premeasurement) and 11.0% (postmeasurement); stop‐signal 2.8% (premeasurement) and 0.9% (postmeasurement) trials).

The effectiveness of the PPI on emotional functioning was tested with several ancovas with condition (PPI vs. WLC) as between‐subjects factor and baseline (premeasurement) scores of the emotional variables (mean centred) as covariates. The intervention is successful in increasing positive and decreasing negative states and traits when results show a significant main effect of condition. This method of analysing is more powerful and precise than using repeated measures anova in a randomized prepost design (Van Breukelen, 2006).

Similar, the effectiveness of the PPI on executive functioning was tested with several ancovas with condition (PPI vs. WLC) as between‐subjects factor and baseline (premeasurement) scores of the executive functioning variables (mean centred) as covariates. The intervention is successful in improving executive task performance (i.e. updating, shifting and inhibition) when results show a significant main effect of condition. Additionally, regression analyses were used to test whether improvements in positive affect and optimism are significant predictors of task performance improvement.

## 3 Results

### 3.1 Baseline descriptives

In total, 33 patients (PPI *n *=* *18, 24.7%; WLC *n *=* *15, 31.3%) did not complete the intervention and/or did not complete all the measurements within the 2 pre‐ and post‐treatment sessions. In the analyses, we included every available data point, resulting in different sample sizes per task/questionnaire (range *n* = 87–121).

We checked for outliers on the general outcome variables (i.e. shifting switch cost RT and accuracy, WCST score, reversed digit span score and stop‐signal SSRT score), with outlier values defined as 3 × SD below or above the overall mean (i.e. cut‐off values). Outlier scores were replaced with these cut‐off values. Additionally, patients that obtained an accuracy score below chance level on the shifting task (<53% correct, as determined by a binomial calculator) were omitted from further analysis for that specific task (*n *=* *2, 2.25%).

Results of the independent‐samples *t*‐tests and chi‐square tests revealed no significant differences between the PPI and WLC condition at baseline (emotional functioning all *p*‐values >0.12; executive functioning all *p*‐values >0.30; patient characteristics all *p*‐values >0.21). See Supporting Information Table S2 for patient characteristics per condition.

Results of the logistic regression analyses indicated that dropout was predicted by lower education level (*B *=* *−0.08, *t* (95) = −3.37, *p *<* *0.01, 95% CI of *B* [−0.125 to −0.032]), more self‐reported ability to live desired life despite pain (*B *=* *0.01, *t* (95) = 2.99, *p *<* *0.01, 95% CI of *B* [0.002 to 0.010]) and more negative future expectancies (*B *=* *0.01, *t* (95) = 2.19, *p *=* *0.03, 95% CI of *B* [0.001 to 0.025]). A significant proportion of variance was explained with, *R*² = 0.28, *F* (18, 95) = 2.04, *p *=* *0.01. All other baseline characteristics, premeasurements and condition were not significant predictors (all *p*'s *>* 0.11*)*. The main reasons that were given to discontinue with the intervention were the start of a treatment trajectory, relationship problems, hospitalization, death of a loved one and not enough time to spend on the intervention.

### 3.2 Emotional and physical functioning

The ancovas revealed a significant main effect of PPI condition, controlling for the effect of scores obtained before the intervention, for happiness (*F* (1, 90) = 4.58, *p *=* *0.04, ηp² = 0.05), optimism (*F* (1, 86) = 7.34, *p *=* *0.01, ηp² = 0.08), positive future expectancies (*F* (1, 86) = 15.15, *p *<* *0.001, ηp² = 0.15), positive affect (*F* (1, 86 = 5.47, *p *=* *0.02, ηp² = 0.06), self‐compassion (*F* (1, 86 = 12.18, *p *>* *0.01, ηp² = 0.12), pain catastrophizing (*F* (1, 86 = 13.92, *p *>* *0.001, ηp² = 0.14), depression (*F* (1, 86 = 6.36, *p *=* *0.01, ηp² = 0.07), anxiety (*F* (1, 86 = 6.21, *p *=* *0.02, ηp² = 0.07) and ability to live a desired life despite pain (*F* (1, 89 = 5.68, *p *=* *0.02, ηp² = 0.06). The PPI condition main effect was not significant for negative future expectancies (*F* (1, 86) = 3.78, *p *=* *0.06, ηp² = 0.04), negative affect (*F* (1, 86) = 2.70, *p *=* *0.10, ηp² = 0.03), pain disability (*F* (1, 86) = 1.31, *p *=* *0.26, ηp² = 0.02) and pain intensity (*F* (1, 89) = 2.05, *p *=* *0.16, ηp² = 0.02). The pre‐ and postscores on the emotional functioning variables are displayed in Table 1. Patients in the PPI condition scored higher on happiness, optimism, positive future expectancies, positive affect, self‐compassion and ability to live a desired life despite pain, compared to patients in the WLC condition. In contrast, patients in the PPI condition scored lower on pain catastrophizing, depression and anxiety compared to patients in the WLC condition.

**Table 1:** Mean (M) and standard deviation (SD) scores on the emotional functioning variables for the pre‐ and postmeasurement, displayed per condition

	Positive psychology intervention			Waiting list control		
	*n*	Premeasurement M (SD)	Postmeasurement M (SD)	*n*	Premeasurement M (SD)	Postmeasurement M (SD)
Positive traits						
Happiness	57	3.84 (0.94)	4.35 (0.83)	36	3.78 (0.80)	3.94 (0.98)
Optimism	56	19.91 (3.95)	21.80 (3.92)	33	20.06 (4.12)	20.21 (3.29)
Self‐compassion	56	52.36 (13.42)	57.48 (11.69)	33	48.82 (13.82)	48.82 (11.35)
Positive states						
Positive affect	56	26.96 (7.33)	28.55 (7.52)	33	25.88 (6.69)	24.58 (7.45)
Positive future expectancies	56	41.77 (10.41)	47.54 (10.30)	33	42.76 (11.58)	41.91 (9.91)
Negative traits						
Pain catastrophizing	56	19.00 (10.48)	12.66 (7.91)	33	20.58 (10.23)	18.85 (8.98)
Negative states						
Anxiety	56	8.55 (4.10)	7.04 (3.54)	33	8.82 (3.84)	8.70 (3.50)
Depression	56	6.36 (3.98)	5.04 (3.45)	34	6.67 (3.88)	6.88 (4.09)
Negative affect	56	18.39 (7.09)	16.50 (6.81)	33	17.97 (7.04)	18.39 (6.84)
Negative future expectancies	56	36.27 (9.93)	32.86 (9.48)	33	39.00 (10.21)	37.76 (9.88)
Pain variables						
Pain disability	56	36.36 (23.29)	33.21 (13.17)	33	38.79 (12.83)	37.45 (12.87)
Ability to live a desired life despite pain	57	41.37 (23.21)	55.18 (22.32)	35	42.49 (24.32)	46.43 (21.84)
Pain intensity	57	60.81 (23.52)	55.12 (23.22)	35	70.46 (14.69)	65.71 (20.63)

### 3.3 Executive functioning

No effect of PPI condition was found for any of the executive functioning tasks (reversed digit span (*F* (1, 85) = 0.33, *p *=* *0.33, ηp² < 0.01; RT switch cost (*F* (1, 84) = 0.28, *p *=* *0.60, ηp² < 0.01; switch cost accuracy (*F* (1, 84) = 0.03, *p *=* *0.86, ηp² < 0.001; WCST score (*F* (1, 88) = 0.21, *p *=* *0.65, ηp² < 0.01); stop‐signal SSRT score (*F* (1, 87) = 0.02, *p *=* *0.90, ηp² < 0.001). Mean and standard deviation scores of the executive task performance variables are displayed in Table 2. We repeated the analyses on the executive functioning dependent variables with pain intensity, age and medication use (yes vs. no) as covariates; again the condition effect was not significant (all *p*‐values > 0.06). To examine a possible learning effect, we conducted several paired‐samples *t*‐tests. The WCST (*t* (90) = 3.29, *p *<* *0.01, *d *=* *0.69, 95% CI of *d* [0.39 to 0.99]) and the stop‐signal task (*t* (89) = 2.97, *p *<* *0.01, *d *=* *0.63, 95% CI of *d* [0.33 to 0.93]) showed significant practice effects (all other *p*‐values > 0.13).

**Table 2:** Mean (M), standard deviation (SD) scores on the executive functioning variables for the pre‐ and postmeasurement, displayed per condition

	Positive psychology intervention (PPI)			Waiting list control (WLC)		
	*n*	Premeasurement M(SD)	Postmeasurement M (SD)	*n*	Premeasurement M (SD)	Postmeasurement M (SD)
Updating						
Reverse digit span	55	29.13 (21.85)	31.39 (21.12)	33	28.07 (23.22)	33.56 (26.61)
Shifting						
Switch cost						
Reaction time (ms)	55	175.31 (257.97)	172.42 (200.06)	32	140.92 (162.59)	140.12 (184.61)
Accuracy (sum)	55	1.67 (3.91)	2.50 (3.26)	32	1.69 (4.26)	2.38 (3.65)
WCST	57	5.04 (3.21)	6.05 (3.08)	34	5.06 (3.24)	5.82 (3.55)
Inhibition						
Stop‐signal SSRT	56	397.66 (91.14)	378.48 (58.25)	34	404.17 (58.93)	379.61 (47.82)

Reverse digit span score = correctly recalled digits; switch cost reaction time = reaction time switch trial minus reaction time repeat trial (ms); switch cost accuracy = accuracy repeat trial minus accuracy switch trial; WCST score = sum categories completed; stop‐signal SSRT score = mean RT on correct go‐trial minus mean stop‐signal delay.

We examined with regression analyses whether improvements in positive affect and optimism were significantly related to improvements in task performance, irrespective of condition. No consistent associations between changes in either positive affect or optimism and improvements in task performance were found. Additionally, we examined whether the task experience (i.e. fatiguing, difficulty, level of pain during task and ability to concentrate on task) changed after the PPI compared to WLC. None of the time (premeasurement vs. postmeasurement) × condition interaction effects were significant (all *p*‐values > 0.08).

## 4 Discussion

This study was set up as a clinical experiment to examine whether an online positive psychology intervention can improve executive functioning in chronic pain patients. As expected, the intervention led to increases in positive emotions and optimism compared to a waiting list control condition. However, the results did not confirm that executive task performance improved in patients that completed the intervention, compared to patients in the waiting list condition.

Based on theoretical and empirical evidence (Fredrickson, 2004; Legrain et al., 2009; Boselie et al., 2014, 2017), we hypothesized that increasing optimism and positive emotions would protect against executive functioning impairments due to chronic pain. Drawn from the broaden‐and‐build theory (Fredrickson, 2001), positive affect may (re) direct an individual's attention (i.e. the broaden hypothesis) towards accurate task performance and/or increase cognitive resources (i.e. the build hypothesis). Within the context of the competing limited resources theory (Eccleston and Crombez, 1999), two potential mechanisms are probable. First, cognitive and self‐regulatory resources are considered to be limited and proposed to be fatigued by pain (Eccleston and Crombez, 1999; Solberg Nes et al., 2010). Experiencing positive emotions and optimism may increase motivation to continue allocating resources towards task performance, despite less resources being available. Second, optimism and positive emotions may increase an individual's ability to preserve attention for accurate task performance. Chronic pain patients often worry and ruminate about their pain experience and report more pain catastrophizing (Eccleston et al., 2001; Kane et al., 2007). As optimism is associated with less worry, rumination and pain catastrophizing (Goodin et al., 2013; Tucker et al., 2013; Bostick et al., 2017) all available attention resources can be dedicated towards task performance instead of being consumed by negative thought patterns. Furthermore, positive emotions and optimism may increase top‐down variables (e.g. goal perseverance and effort (Van Damme et al., 2010; Wrosch and Scheier, 2003) that modulate the ability of pain to interrupt and capture an individual's attention at the expense of accurate task performance (Legrain et al., 2009). Besides these theoretical underpinnings, results from two laboratory studies (Boselie et al., 2014, 2017) demonstrated that increasing positive emotions and optimism, by means of a writing and visualisation exercise, counteracted pain‐induced deteriorations in executive task performance (i.e. updating and shifting) in healthy university students.

However, the current study findings indicate that these previously found beneficial effects of optimism on executive functioning in healthy participants do not extend to chronic pain patients. Despite the fact that the intervention successfully increased positive emotions and optimism, this increase did not translate in improvements in executive task performance. Given that there are fundamental differences between experimentally induced pain and natural occurring persistent pain (i.e. less predictable, persistent and more threatening Edens and Gil, 1995), it is conceivable that positive emotions and optimism cannot improve executive functioning in the context of chronic pain. However, there are also several reasons that could account for these different findings. First, the cognitive deficits in chronic pain patients may be too pervasive and longstanding to be ameliorated by the intervention. Second, we theorized that optimism may increase motivation to continue allocating resources towards accurate executive task performance, despite less resources being available due to pain. But this increased motivation by optimism may only occur, or may especially occur, when the task is perceived as important, valuable and personally relevant (Van Damme et al., 2010). Future studies are necessary to elucidate the importance of task relevance. Third, in contrast to a patient sample, a healthy student sample is more homogeneous, the age range is more restricted and students are generally more familiar with performing computer tasks that measure executive functioning. Furthermore, we included extended practice phases, elaborated task instruction and showed the instructions during task execution to ensure good comprehension of the task by patients. However, these measures may not compensate for lack of computer experience or anxiety for completing computerized tests, which negatively influence task performance (Browndyke et al., 2002). Lastly, methodological differences between the studies could also have played a role. Although delivering an intervention via Internet has several advantages, such as high accessibility and lower threshold to participate (Hedman et al., 2012; Andersson and Titov, 2014), disadvantages to the online data collection can be identified as well. For example, the online mode of administration restricted the possibility to give clarification about the tasks. Furthermore, although we applied rigorous data screening methods, it cannot be excluded that task performance was affected by test environment or that patients did not dedicate the appropriate amount of effort, resulting in poorer task performance (Attridge et al., 2016). Additionally, to measure improvement in executive functioning, the same tasks were used for the pre‐ and postmeasurement. However, performing a task twice often leads to improved task performance (i.e. practice effects Falleti et al., 2006), which may have masked the effects of the intervention on executive functioning. Future research may consider implementing a task twice at baseline to minimize practice effects, the influence of computer experience and task incomprehension. This gives patients the time to fully grasp the requirements of the different tasks, increasing the likelihood that executive functioning is measured, not mere task comprehension (Falleti et al., 2006).

A further difficulty is that performance on experimental tasks may have little predictive value for how a patient will perform on naturalistic tasks encountered in everyday life (Chan et al., 2008). For example, patients may be perfectly able to switch between sorting rules in the WCST, but unable to switch between different languages in a conversation, although both require shifting ability. To overcome this lack of predictive value, we need more ecological valid measurements of executive functioning. Proposed solutions are developing tasks based on themes essential for survival (e.g. increasing social cooperation, parenting), increasing complexity (i.e. multiple steps to obtain one overarching goal, such as driving or cooking), making the task more personal relevant, or even using virtual reality to mimic complex real‐world situations (Chan et al., 2008; Keogh et al., 2013).

We did replicate the findings of a previously conducted randomized control trial (Peters et al., 2017) in that the intervention was successful in increasing happiness, optimism, positive future expectancies, positive affect, self‐compassion and ability to live a desired life despite pain, and lowering pain catastrophizing, depression and anxiety. These findings emphasize the possibility to increase emotional well‐being and decrease distress in chronic pain patients through an online self‐help positive psychology intervention. The intervention did not decrease pain intensity and disability, which is not an uncommon outcome when examining psychological treatments for chronic pain (Eccleston et al., 2009). That pain persists validates the importance of having self‐help techniques to reduce the burden of living with chronic pain. Moreover, these significant improvements were achieved via minimal guidance, making it an attractive treatment option for chronic pain patients.

We should note that the generalization of these results towards other pain disorders or patients in other settings is limited as the study sample consisted of predominately self‐reported fibromyalgia patients who self‐selected to participate in this study. However, prior research has indicated that fibromyalgia patients have poorer treatment response to CBT than other pain disorders (Sturgeon, 2014), thus finding a successful intervention for this pain disorder is imperative. Nevertheless, future research is necessary to examine whether these findings extend to other pain disorders or settings. Another future direction could be to examine whether the PPI intervention can be more effective when the intervention is personalized (Ritterband et al., 2003) or whether combining the PPI with other interventions (e.g. exposure, CBT) has additional benefits. Furthermore, the intervention consisted of several evidence‐based positive psychology exercises, but it is unclear what the distinctive effect of each technique was on treatment outcome. Similarly, adding adherence assessments (e.g. login data) and subjective evaluations of the intervention could improve the intervention further.

Future studies may consider having intermediate measurements following the completion of every module so it is possible to examine the distinctive effect of each module. Additionally, a diary assessment could be added to assess the impact of the PPI on a patient's daily life functioning in more depth.

In conclusion, the present study shows that an 8‐week online self‐help positive psychology intervention is able to improve emotional well‐being in chronic pain patients. However, the intervention did not improve executive task performance. We advocate to further test whether the protective effect of optimism on executive functioning can be extended to chronic pain patients. Future research could for instance measure executive task performance under more controlled circumstances (i.e. in the laboratory), use more ecologically valid tasks (e.g. more complex, increasing personal relevance and virtual reality) and use multiple baselines measurements of executive functioning. Despite the lack of evidence that positive emotions and optimism can improve executive task performance in chronic pain patients, this study did convincingly demonstrate that it is possible to increase positive emotions and optimism in chronic pain patients with an online positive psychology intervention. As pain itself often cannot be relieved, it is imperative to have techniques to reduce the burden of living with chronic pain.

## Author contributions

All authors have contributed to the conception and design of the study and discussed the results and commented on the manuscript. The first author also acquired and analysed the data.

## Supporting information


**Figure S1** Overview of the PPI modules.

**Figure S2** Timeline of events in the task shifting paradigm, showing two consecutive runs of trials.

**Figure S3** Example of the sorting rules in the Wisconsin card sorting task. Note: colour is not an accurate depiction of the colour used in the task.

**Table S1** Inclusion and exclusion criteria.

**Table S2** Patient characteristics, displayed per condition.

## References

[ejp1214-bib-0001] Abdel‐Khalek, A.M. (2006). Measuring happiness with a single‐item scale. Soc Behav Pers 34, 139–150.

[ejp1214-bib-0002] Altmann, E.M. , Gray, W.D. (2008). An integrated model of cognitive control in task switching. Psychol Rev 115, 602. 18729594 10.1037/0033-295X.115.3.602

[ejp1214-bib-0003] Andersson, G. , Titov, N. (2014). Advantages and limitations of Internet‐based interventions for common mental disorders. World Psychiatry 13, 4–11. 24497236 10.1002/wps.20083 PMC3918007

[ejp1214-bib-0004] Attridge, N. , Keogh, E. , Eccleston, C. (2016). The effect of pain on task switching: Pain reduces accuracy and increases reaction times across multiple switching paradigms. Pain 157, 2179–2193. 27513452 10.1097/j.pain.0000000000000627

[ejp1214-bib-0503] Banich, M.T. (2009). Executive function the search for an integrated account. Curr Dir Psychol Sci 18, 89–94.

[ejp1214-bib-0005] Benedict, R.H. , Zgaljardic, D.J. (1998). Practice effects during repeated administrations of memory tests with and without alternate forms. J Clin Exp Neuropsychol 20, 339–352. 9845161 10.1076/jcen.20.3.339.822

[ejp1214-bib-0006] Berryman, C. , Stanton, T.R. , Bowering, K.J. , Tabor, A. , McFarlane, A. , Moseley, G.L. (2013). Evidence for working memory deficits in chronic pain: A systematic review and meta‐analysis. Pain 154, 1181–1196. 23707355 10.1016/j.pain.2013.03.002

[ejp1214-bib-0007] Berryman, C. , Stanton, T.R. , Bowering, K.J. , Tabor, A. , McFarlane, A. , Moseley, G.L. (2014). Do people with chronic pain have impaired executive function? A meta‐analytical review Clin Psychol Rev 34, 563–579. 25265056 10.1016/j.cpr.2014.08.003

[ejp1214-bib-0008] Bialocerkowski, A.E. (2008). Measurement error and reliability testing: Application to rehabilitation. Int J Ther Rehabil 15, 422–427.

[ejp1214-bib-0009] Boselie, J.J. , Vancleef, L.M. , Smeets, T. , Peters, M.L. (2014). Increasing optimism abolishes pain‐induced impairments in executive task performance. Pain 155, 334–340. 24145210 10.1016/j.pain.2013.10.014

[ejp1214-bib-0010] Boselie, J.J. , Vancleef, L.M. , Peters, M.L. (2016). The effects of experimental pain and induced optimism on working memory task performance. Scand J Pain 12, 25–32. 28850487 10.1016/j.sjpain.2016.03.001

[ejp1214-bib-0011] Boselie, J.J. , Vancleef, L.M. , Peters, M.L. (2017). Increasing optimism protects against pain‐induced impairment in task‐shifting performance. J Pain 18, 446–455. 28039101 10.1016/j.jpain.2016.12.007

[ejp1214-bib-0012] Bostick, G. , Kamper, S. , Haanstra, T. , Dick, B. , Stitt, L. et al. (2017). Pain expectations in neuropathic pain: Is it best to be optimistic? Eur J Pain 21, 605–613. 27739623 10.1002/ejp.962

[ejp1214-bib-0013] Breivik, H. , Collett, B. , Ventafridda, V. , Cohen, R. , Gallacher, D. (2006). Survey of chronic pain in Europe: Prevalence, impact on daily life, and treatment. Eur J Pain 10, 287–333. 16095934 10.1016/j.ejpain.2005.06.009

[ejp1214-bib-0014] Browndyke, J.N. , Albert, A.L. , Malone, W. , Schatz, P. , Paul, R.H. , Cohen, R.A. , Tucker, K.A. , Gouvier, W.D. (2002). Computer‐related anxiety: Examining the impact of technology‐specific affect on the performance of a computerized neuropsychological assessment measure. Appl Neuropsychol 9, 210–218. 12584075 10.1207/S15324826AN0904_3

[ejp1214-bib-0015] Buhle, J. , Wager, T.D. (2010). Performance‐dependent inhibition of pain by an executive working memory task. Pain 149, 19–26. 20129735 10.1016/j.pain.2009.10.027 PMC4229048

[ejp1214-bib-0501] Carver, C.S. , Scheier, M.F. , Segerstrom, S.C. (2010). Optimism. Clin Psychol Rev 30, 879–889. 20170998 10.1016/j.cpr.2010.01.006 PMC4161121

[ejp1214-bib-0016] Chan, R.C. , Shum, D. , Toulopoulou, T. , Chen, E.Y. (2008). Assessment of executive functions: Review of instruments and identification of critical issues. Arch Clin Neuropsychol 23, 201–216. 18096360 10.1016/j.acn.2007.08.010

[ejp1214-bib-0017] Crawford, J.R. , Henry, J.D. (2004). The Positive and Negative Affect Schedule (PANAS): Construct validity, measurement properties and normative data in a large non‐clinical sample. Br J Clin Psychol 43, 245–265. 15333231 10.1348/0144665031752934

[ejp1214-bib-0018] Eccleston, C. , Crombez, G. (1999). Pain demands attention: A cognitive–affective model of the interruptive function of pain. Psychol Bull 125, 356–366. 10349356 10.1037/0033-2909.125.3.356

[ejp1214-bib-0019] Eccleston, C. , Crombez, G. , Aldrich, S. , Stannard, C. (2001). Worry and chronic pain patients: A description and analysis of individual differences. Eur J Pain 5, 309–318. 11558986 10.1053/eujp.2001.0252

[ejp1214-bib-0020] Eccleston, C. , ACdC, W. , Morley, S. (2009). Psychological therapies for the management of chronic pain (excluding headache) in adults. *The cochrane library*. 10.1002/14651858.CD007407.pub2 19370688

[ejp1214-bib-0021] Edens, J.L. , Gil, K.M. (1995). Experimental induction of pain: Utility in the study of clinical pain. Behav Ther 26, 197–216.

[ejp1214-bib-0022] Falleti, M.G. , Maruff, P. , Collie, A. , Darby, D.G. (2006). Practice effects associated with the repeated assessment of cognitive function using the CogState battery at 10‐minute, one week and one month test‐retest intervals. J Clin Exp Neuropsychol 28, 1095–1112. 16840238 10.1080/13803390500205718

[ejp1214-bib-0023] Faul, F. , Erdfelder, E. , Lang, A.‐G. , Buchner, A. (2007). G* Power 3: A flexible statistical power analysis program for the social, behavioral, and biomedical sciences. Behav Res Methods 39, 175–191. 17695343 10.3758/bf03193146

[ejp1214-bib-0024] Fredrickson, B.L. (2001). The role of positive emotions in positive psychology: The broaden‐and‐build theory of positive emotions. Am Psychol 56, 218. 11315248 10.1037//0003-066x.56.3.218 PMC3122271

[ejp1214-bib-0025] Fredrickson, B.L. (2004). The broaden‐and‐build theory of positive emotions. Phil Trans R Soc Lond Ser B Biol Sci 359, 1367–1378. 15347528 10.1098/rstb.2004.1512 PMC1693418

[ejp1214-bib-0502] Gilbert, S.J. , Burgess, P.W. (2008). Executive function. Curr Biol 18, R110–R114. 18269902 10.1016/j.cub.2007.12.014

[ejp1214-bib-0026] Goodin, B.R. , Glover, T.L. , Sotolongo, A. , King, C.D. , Sibille, K.T. et al. (2013). The association of greater dispositional optimism with less endogenous pain facilitation is indirectly transmitted through lower levels of pain catastrophizing. J Pain 14, 126–135. 23218934 10.1016/j.jpain.2012.10.007 PMC3592576

[ejp1214-bib-0027] Goubert, L. , Trompetter, H. (2017). Towards a science and practice of resilience in the face of pain. Eur J Pain 21, 1301–1315. 28573783 10.1002/ejp.1062

[ejp1214-bib-0028] Greve, K.W. , Stickle, T.R. , Love, J.M. , Bianchini, K.J. , Stanford, M.S. (2005). Latent structure of the Wisconsin Card Sorting Test: A confirmatory factor analytic study. Arch Clin Neuropsychol 20, 355–364. 15797171 10.1016/j.acn.2004.09.004

[ejp1214-bib-0029] Hanssen, M.M. , Peters, M.L. , Vlaeyen, J.W. , Meevissen, Y.M. , Vancleef, L.M. (2013). Optimism lowers pain: Evidence of the causal status and underlying mechanisms. Pain 154, 53–58. 23084002 10.1016/j.pain.2012.08.006

[ejp1214-bib-0504] Hart, R.P. , Martelli, M.F. , Zasler, N.D. (2000). Chronic pain and neuropsychological functioning. Neuropsychol Rev 10, 131–149. 10983898 10.1023/a:1009020914358

[ejp1214-bib-0030] Heaton, R. , Chelune, G. , Talley, J. , Kay, G. , Curtiss, G. (1993). Wisconsin Card Sorting Test Manual Revised and Expanded. (Lutz, FL: Psychological Assessment Resources. Inc).

[ejp1214-bib-0031] Hedman, E. , Ljótsson, B. , Lindefors, N. (2012). Cognitive behavior therapy via the Internet: A systematic review of applications, clinical efficacy and cost–effectiveness. Expert Rev Pharmacoecon Outcomes Res 12, 745–764. 23252357 10.1586/erp.12.67

[ejp1214-bib-0032] Kane, M.J. , Brown, L.H. , McVay, J.C. , Silvia, P.J. , Myin‐Germeys, I. , Kwapil, T.R. (2007). For whom the mind wanders, and when: An experience‐sampling study of working memory and executive control in daily life. Psychol Sci 18, 614–621. 17614870 10.1111/j.1467-9280.2007.01948.x

[ejp1214-bib-0033] Keogh, E. , Moore, D.J. , Duggan, G.B. , Payne, S.J. , Eccleston, C. (2013). The disruptive effects of pain on complex cognitive performance and executive control. PLoS ONE 8, e83272. 24386168 10.1371/journal.pone.0083272 PMC3875458

[ejp1214-bib-0034] Keogh, E. , Cavill, R. , Moore, D.J. , Eccleston, C. (2014). The effects of menstrual‐related pain on attentional interference. Pain 155, 821–827. 24486337 10.1016/j.pain.2014.01.021

[ejp1214-bib-0035] Kiesel, A. , Steinhauser, M. , Wendt, M. , Falkenstein, M. , Jost, K. , Philipp, A.M. , Koch, I. (2010). Control and interference in task switching—A review. Psychol Bull 136, 849. 20804238 10.1037/a0019842

[ejp1214-bib-0036] Korn, E.L. , Freidlin, B. (2011). Outcome‐adaptive randomization: Is it useful? J Clin Oncol 29, 771–776. 21172882 10.1200/JCO.2010.31.1423 PMC3056658

[ejp1214-bib-0037] Kowal, J. , Wilson, K.G. , McWilliams, L.A. , Péloquin, K. , Duong, D. (2012). Self‐perceived burden in chronic pain: Relevance, prevalence, and predictors. Pain 153, 1735–1741. 22703692 10.1016/j.pain.2012.05.009 PMC3999031

[ejp1214-bib-0038] Legrain, V. , Damme, S.V. , Eccleston, C. , Davis, K.D. , Seminowicz, D.A. , Crombez, G. (2009). A neurocognitive model of attention to pain: Behavioral and neuroimaging evidence. Pain 144, 230–232. 19376654 10.1016/j.pain.2009.03.020

[ejp1214-bib-0039] Miyake, A. , Friedman, N.P. , Emerson, M.J. , Witzki, A.H. , Howerter, A. (2000). The unity and diversity of executive functions and their contributions to complex ‘frontal lobe’ tasks: A latent variable analysis. Cogn Psychol 41, 49–100. 10945922 10.1006/cogp.1999.0734

[ejp1214-bib-0040] Moore, D.J. , Keogh, E. , Eccleston, C. (2012). The interruptive effect of pain on attention. Q J Exp Psychol 65, 565–586. 10.1080/17470218.2011.626865 22136653

[ejp1214-bib-0041] Moore, D.J. , Keogh, E. , Eccleston, C. (2013). Headache impairs attentional performance. Pain 154, 1840–1845. 23748118 10.1016/j.pain.2013.06.006

[ejp1214-bib-0042] Moriarty, O. , McGuire, B.E. , Finn, D.P. (2011). The effect of pain on cognitive function: A review of clinical and preclinical research. Prog Neurobiol 93, 385–404. 21216272 10.1016/j.pneurobio.2011.01.002

[ejp1214-bib-0505] Morris, N. , Jones, D.M. (1990). Memory updating in working memory: The role of the central executive. Br J Psychol 81, 111–121.

[ejp1214-bib-0043] Neff, K.D. (2003). The development and validation of a scale to measure self‐compassion. Self Identity 2, 223–250.

[ejp1214-bib-0044] Neff, K.D. , Germer, C.K. (2013). A pilot study and randomized controlled trial of the mindful self‐compassion program. J Clin Psychol 69, 28–44. 23070875 10.1002/jclp.21923

[ejp1214-bib-0045] Peters, M.L. , Flink, I.K. , Boersma, K. , Linton, S.J. (2010). Manipulating optimism: Can imagining a best possible self be used to increase positive future expectancies? J Posit Psychol 5, 204–211.

[ejp1214-bib-0046] Peters, M.L. , Smeets, E. , Feijge, M. , vanBreukelen, G. , Andersson, G. , Buhrman, M. , Linton, S.J. (2017). Happy despite pain: A randomized controlled trial of an 8‐week internet‐delivered positive psychology intervention for enhancing well‐being in patients with chronic pain. Clin J Pain 33, 962. 28379873 10.1097/AJP.0000000000000494 PMC5636048

[ejp1214-bib-0047] Pollard, C.A. (1984). Preliminary validity study of the pain disability index. Percept Mot Skills 59, 974. 6240632 10.2466/pms.1984.59.3.974

[ejp1214-bib-0048] Raes, F. , Pommier, E. , Neff, K.D. , Van Gucht, D. (2011). Construction and factorial validation of a short form of the self‐compassion scale. Clin Psychol Psychother 18, 250–255. 21584907 10.1002/cpp.702

[ejp1214-bib-0049] Ritterband, L.M. , Gonder‐Frederick, L.A. , Cox, D.J. , Clifton, A.D. , West, R.W. , Borowitz, S.M. (2003). Internet interventions: In review, in use, and into the future. Prof Psychol Res Pract 34, 527–534.

[ejp1214-bib-0050] Scheier, M.F. , Carver, C.S. , Bridges, M.W. (1994). Distinguishing optimism from neuroticism (and trait anxiety, self‐mastery, and self‐esteem): A reevaluation of the Life Orientation Test. J Pers Soc Psychol 67, 1063–1078. 7815302 10.1037//0022-3514.67.6.1063

[ejp1214-bib-0051] Seligman, M.E. , Rashid, T. , Parks, A.C. (2006). Positive psychotherapy. Am Psychol 61, 774. 17115810 10.1037/0003-066X.61.8.774

[ejp1214-bib-0052] Severeijns, R. , van den Hout, M.A. , Vlaeyen, J.W. , Picavet, H.S.J. (2002). Pain catastrophizing and general health status in a large Dutch community sample. Pain 99, 367–376. 12237216 10.1016/s0304-3959(02)00219-1

[ejp1214-bib-0053] Solberg Nes, L. , Carlson, C.R. , Crofford, L.J. , de Leeuw, R. , Segerstrom, S.C. (2010). Self‐regulatory deficits in fibromyalgia and temporomandibular disorders. Pain 151, 37–44. 20561749 10.1016/j.pain.2010.05.009

[ejp1214-bib-0054] Spinhoven, P. , Ormel, J. , Sloekers, P. , Kempen, G. , Speckens, A. , Van Hemert, A. (1997). A validation study of the Hospital Anxiety and Depression Scale (HADS) in different groups of Dutch subjects. Psychol Med 27, 363–370. 9089829 10.1017/s0033291796004382

[ejp1214-bib-0055] Stewart, W.F. , Ricci, J.A. , Chee, E. , Morganstein, D. , Lipton, R. (2003). Lost productive time and cost due to common pain conditions in the US workforce. JAMA 290, 2443–2454. 14612481 10.1001/jama.290.18.2443

[ejp1214-bib-0056] Sturgeon, J.A. (2014). Psychological therapies for the management of chronic pain. Psychol Res Behav Manag 7, 115. 24748826 10.2147/PRBM.S44762 PMC3986332

[ejp1214-bib-0057] Sullivan, M.J.L. , Bishop, S.R. , Pivik, J. (1995). The pain catastrophizing scale: Development and validation. Psychol Assess 7, 524–532.

[ejp1214-bib-0058] Tait, R.C. , Chibnall, J.T. , Krause, S. (1990). The pain disability index: Psychometric properties. Pain 40, 171–182. 2308763 10.1016/0304-3959(90)90068-O

[ejp1214-bib-0059] Tien, A.Y. , Spevack, T.V. , Jones, D.W. , Pearlson, G.D. , Schlaepfer, T.E. , Strauss, M.E. (1996). Computerized Wisconsin card sorting test: Comparison with manual administration. Kaohsiung J Med Sci 12, 479–485. 8774117

[ejp1214-bib-0060] Tucker, R.P. , Wingate, L.R. , O'Keefe, V.M. , Mills, A.C. , Rasmussen, K. , Davidson, C.L. , Grant, D.M. (2013). Rumination and suicidal ideation: The moderating roles of hope and optimism. Personality Individ Differ 55, 606–611.

[ejp1214-bib-0061] Van Breukelen, G.J.P. (2006). ANCOVA versus change from baseline had more power in randomized studies and more bias in nonrandomized studies. J Clin Epidemiol 59, 920–925. 16895814 10.1016/j.jclinepi.2006.02.007

[ejp1214-bib-0062] Van Damme, S. , Legrain, V. , Vogt, J. , Crombez, G. (2010). Keeping pain in mind: A motivational account of attention to pain. Neurosci Biobehav Rev 34, 204–213. 19896002 10.1016/j.neubiorev.2009.01.005

[ejp1214-bib-0063] Van Ryckeghem, D.M. , Crombez, G. , Eccleston, C. , Liefooghe, B. , Van Damme, S. (2012). The interruptive effect of pain in a multitask environment: An experimental investigation. J Pain 13, 131–138. 22071367 10.1016/j.jpain.2011.09.003

[ejp1214-bib-0064] Verdejo‐García, A. , López‐Torrecillas, F. , Calandre, E.P. , Delgado‐Rodríguez, A. , Bechara, A. (2009). Executive function and decision‐making in women with fibromyalgia. Arch Clin Neuropsychol 24, 113–122. 19395361 10.1093/arclin/acp014

[ejp1214-bib-0065] Watson, D. , Clark, L.A. , Tellegen, A. (1988). Development and validation of brief measures of positive and negative affect: The PANAS scales. J Pers Soc Psychol 54, 1063–1070. 3397865 10.1037//0022-3514.54.6.1063

[ejp1214-bib-0066] Wechsler, D. (1997). WAIS‐III: Administration and scoring manual: Wechsler adult intelligence scale Psychological Corporation.

[ejp1214-bib-0067] Wrosch, C. , Scheier, M.F. (2003). Personality and quality of life: The importance of optimism and goal adjustment. Qual Life Res 12, 59–72. 12803312 10.1023/a:1023529606137

[ejp1214-bib-0068] Zigmond, A.S. , Snaith, R.P. (1983). The hospital anxiety and depression scale. Acta Psychiatr Scand 67, 361–370. 6880820 10.1111/j.1600-0447.1983.tb09716.x

