# Supplementary figures and images for: Filling the glass: Effects of a positive psychology intervention on executive task performance in chronic pain patients
Source: Eur J Pain. 2018 Apr 14;22(7):1268–80. doi: 10.1002/ejp.1214 (PMC6055672; doi:10.1002/ejp.1214)

***
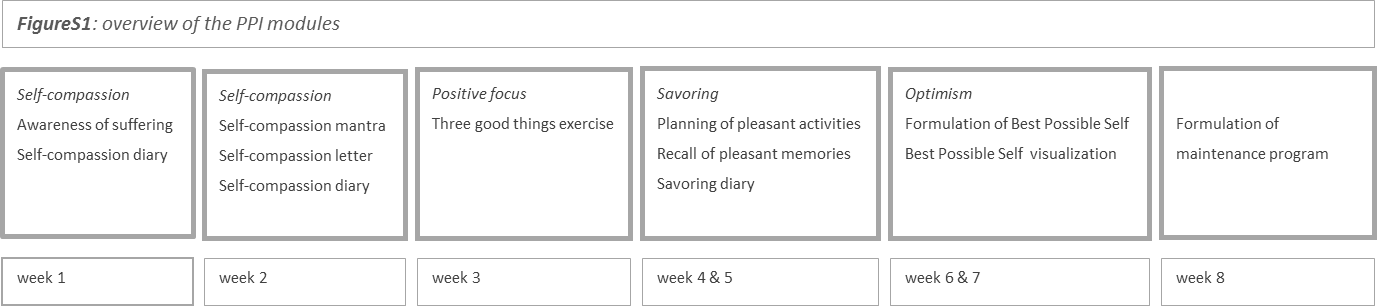
***

Supplement: Supplementary file 1 — Figure S1 Overview of the PPI modules. [file EJP-22-1268-s001.docx]
