# Supplementary material for: Filling the glass: Effects of a positive psychology intervention on executive task performance in chronic pain patients
Source: Eur J Pain. 2018 Apr 14;22(7):1268–80. doi: 10.1002/ejp.1214 (PMC6055672; doi:10.1002/ejp.1214)

***Figure S2.*** Timeline of events in the task shifting paradigm, showing two consecutive runs of trials.


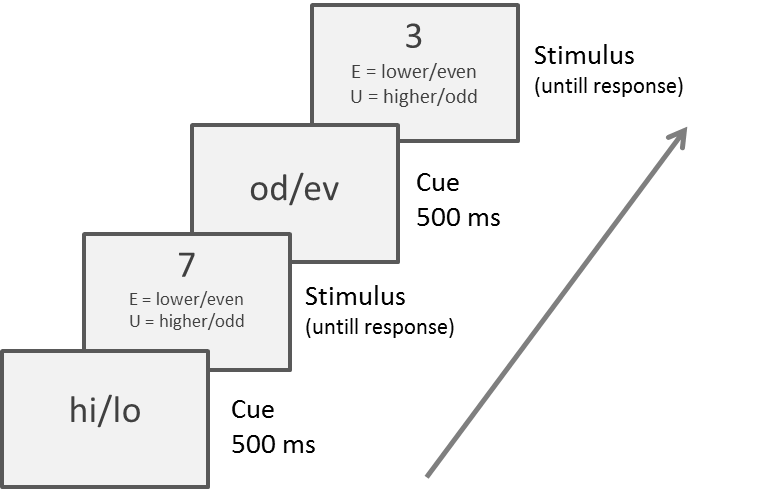

Supplement: Supplementary file 2 — Figure S2 Timeline of events in the task shifting paradigm, showing two consecutive runs of trials. [file EJP-22-1268-s002.docx]
