# Supplementary material for: Filling the glass: Effects of a positive psychology intervention on executive task performance in chronic pain patients
Source: Eur J Pain. 2018 Apr 14;22(7):1268–80. doi: 10.1002/ejp.1214 (PMC6055672; doi:10.1002/ejp.1214)

***Figure S3.*** *Example of the sorting rules in the Wisconsin card sorting task. Note: colour is not an accurate depiction of the colour used in the task.*


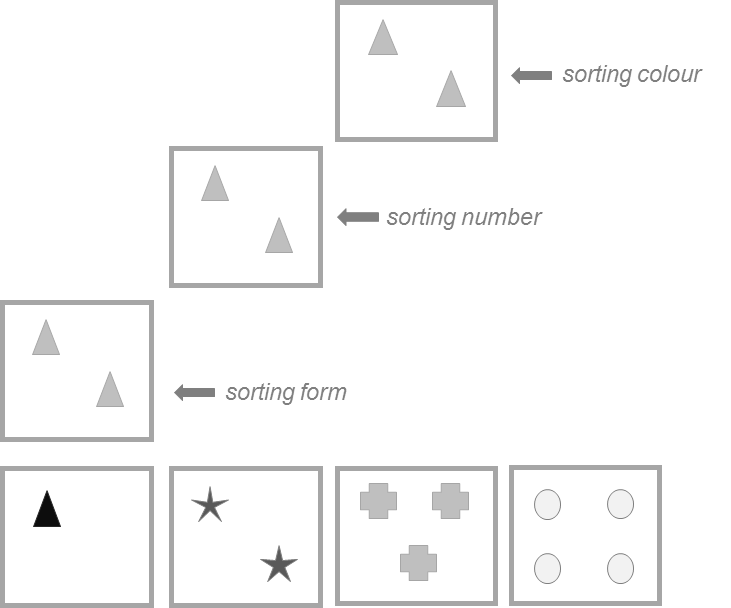

Supplement: Supplementary file 3 — Figure S3 Example of the sorting rules in the Wisconsin card sorting task. Note: colour is not an accurate depiction of the colour used in the task. [file EJP-22-1268-s003.docx]
