# Supplementary material for: Filling the glass: Effects of a positive psychology intervention on executive task performance in chronic pain patients
Source: Eur J Pain. 2018 Apr 14;22(7):1268–80. doi: 10.1002/ejp.1214 (PMC6055672; doi:10.1002/ejp.1214)
Supplement: Supplementary file 4 — Table S1 Inclusion and exclusion criteria. [file EJP-22-1268-s004.docx]

| **TableS1. Inclusion and exclusion criteria** | |
| --- | --- |
| *Inclusion criteria* | *Exclusion criteria* |
| > 18 years | > 60 years |
| Chronic pain lasting longer than 3 months | Psychological or multidisciplinary pain treatment in the past 3 months |
| Having musculoskeletal pain, generalized (*i.e.* Fibromyalgia) or in back, neck or shoulders | Diagnosed with psychopathological disorders in the past 3 months |
| Good mastery Dutch language (also in reading) | Diagnosed with a neurological disorder (such as a stroke or epilepsy) |
| Having access to the Internet | Being pregnant |
| Able to spend 3 hours a week on the program | Not being able to perform tasks on a computer |
|  |  |
