# Supplementary material for: Filling the glass: Effects of a positive psychology intervention on executive task performance in chronic pain patients
Source: Eur J Pain. 2018 Apr 14;22(7):1268–80. doi: 10.1002/ejp.1214 (PMC6055672; doi:10.1002/ejp.1214)
Supplement: Supplementary file 5 — Table S2 Patient characteristics, displayed per condition. [file EJP-22-1268-s005.docx]

| **TableS2.**  **Patient characteristics, displayed per condition.** | | | | |
| --- | --- | --- | --- | --- |
|  | *Positive Psychology Intervention (PPI)*  *n = 73* | *Waiting List*  *Control (WLC)*  *n = 48* | *Total*  *n = 121* |  |
| Age (mean; SD) | 45.59 (9.77) | 43.15 (9.70) | 44.63 (9.82) |  |
| Female (n, %) | 70 (95.9) | 47 (97.9) | 117 (96.7) |  |
| Fibromyalgia (n, %) | 61 (83.6) | 40 (83.3) | 101 (83.5)° |  |
| low | 14 (19.2) | 12 (25.0) | 26 (21.5)°° |  |
| medium | 32 (43.8) | 17 (35.4) | 49 (40.5) |  |
| high | 26 (35.6) | 19 (39.6) | 45 (37.2) |  |
| Pain duration in years (mean; SD) | 13.34 (9.64) | 11.77 (9.00) | 12.70 (9.38)°°° |  |
| Employment (n, %) |  |  |  |  |
| Full-time | 10 (13.7) | 8(16.7) | 18 (14.9) |  |
| Part-time | 22 (30.1) | 21 (43.8) | 44 (36.4) |  |
| Stay-at home parent | 9 (12.3) | 3 (6.3) | 12 (9.9) |  |
| Student | 2 (2.7) | 2 (4.2) | 4 (3.3) |  |
| Sick leave | 1 (1.4) | 1 (2.1) | 2 (1.7) |  |
| Disability pension | 14 (19.2) | 6 (12.5) | 20 (16.5) |  |
| Pension | 5 (6.9) | 5 (10.4) | 10 (8.3) |  |
| Unemployed | 10 (13.7) | 2(4.2) | 12 (9.9) |  |
| °*13 patients did not have a Fibromyalgia diagnosis and 7 patients did not report on diagnosis;* °° *1 patient did not report education level; °°°2 patients did not report pain duration.* | | | | |
